# *Plesiochorus irwinorum* n. sp. (Trematoda: Gorgoderidae) from the urinary bladder of the hawksbill turtle, *Eretmochelys imbricata* (Testudines: Cheloniidae), off the east coast of Australia

**DOI:** 10.1007/s11230-022-10038-4

**Published:** 2022-04-23

**Authors:** Richard D. Corner, Rosemary J. Booth, Scott C. Cutmore

**Affiliations:** 1 School of Biological Sciences, The University of Queensland, Saint Lucia, Queensland 4072 Australia; 2 Australia Zoo Wildlife Hospital, Beerwah, Queensland Australia

## Abstract

*Plesiochorus* Looss, 1901 is a genus of Gorgoderidae infecting the urinary bladders of marine turtles globally. Currently, just two morphologically similar species are recognised, *Plesiochorus cymbiformis* (Rudolphi, 1819) Looss, 1901 and *Plesiochorus elongatus* Pigulevsky, 1953, which have been distinguished by molecular data and subtle morphological differences. Here we describe a new species, *Plesiochorus irwinorum* **n. sp.**, infecting hawksbill turtles (*Eretmochelys imbricata* (L.)), which is primarily distinguished from the other two species of *Plesiochorus* on the basis of ITS2, *cox*1 and 28S sequence data. Morphometric data for specimens examined during this study overlap between *P*. *cymbiformis* and *P*. *irwinorum* **n. sp.** for every measured feature, rendering them functionally cryptic. However, principal components analysis clearly distinguishes the two species. Additionally, we report new specimens of *P*. *cymbiformis*, and provide new sequence data for specimens from Australian loggerhead (*Caretta caretta* (L.)) and hawksbill turtles. There is little understanding of the host-specificity or geographical distribution of the three species of *Plesiochorus*, and it remains possible that some of the previously reported sequences have been attributed to the wrong species.

## Introduction

The Gorgoderidae Looss, 1899 is a relatively large family of trematodes that infect a wide range of freshwater and marine vertebrates as definitive hosts, including amphibians, chondrichthyans, teleosts and reptiles (Campbell, 2008). In chondrichthyans, gorgoderids infect the body and pericardial cavities, whereas those of amphibians, teleosts, and reptiles are typically reported from the urinary bladder or rarely, the gall bladder. Only two genera, *Bicornuata* Pearse, 1949 and *Plesiochorus* Looss, 1901, have been reported from marine turtles. The sole species of the monotypic genus *Bicornuata*, *Bicornuata caretta* Pearse, 1949, infects the gall bladder of *Caretta caretta* (L.). The two known species of *Plesiochorus* infect the urinary bladder of marine turtles. *Plesiochorus cymbiformis* (Rudolphi, 1819) Looss, 1901 is reported from *C*. *caretta*, *Chelonia mydas* (L.), *Eretmochelys imbricata* (L.), and *Lepidochelys olivacea* (Eschscholtz) (see Blair & Limpus, 1982; Santoro & Morales, 2007), whereas *Plesiochorus elongatus* Pigulevsky, 1953 is reported from only *C. caretta* (see Werneck et al., 2019).

*Plesiochorus* has been shown to be a phylogenetically stable genus within the subfamily Anaporrhutinae Looss, 1901, with the two known species forming a well-supported monophyletic clade based on ITS2 data (Werneck et al., 2019). The type species, *P*. *cymbiformis*, was originally described from the urinary bladder of *C*. *caretta* from the Adriatic Sea (Rudolphi, 1819) and has subsequently been reported from off Australia, Brazil, Costa Rica, Cuba, Florida, USA, Gulf of Panama, India, the Mediterranean Sea, Mexico, Morocco, New Guinea, Pakistan, Puerto Rico, and the Red Sea (see Blair & Limpus, 1982; Santoro & Morales, 2007; Werneck et al., 2019). Until recently, *P*. *elongatus*, had been considered a subspecies of *P*. *cymbiformis*, recognised as *Plesiochorus cymbiformis elongatus* Pigulevsky, 1953. However, recent molecular studies provided evidence to suggest that *P*. *elongatus* is a distinct species, known from off the coasts of Brazil and Egypt (Werneck et al., 2019). The morphological differences between *P*. *cymbiformis* and *P*. *elongatus* are subtle; *P*. *elongatus* is reported to have a generally more elongate body than *P*. *cymbiformis*, and uterine coils which are closer to the body margin (Werneck et al., 2019). However, gorgoderids have been reported to exhibit striking morphological variation, naturally and as a result of different fixing methods, leading to difficulties in the delineation of species on the basis of morphology alone (Bakke, 1988; Namuleno & Scholz, 1994; Cutmore et al., 2010).

Here, we report gorgoderids infecting *C*. *caretta* and *E*. *imbricata* from off the east coast of Australia, describe a new species of *Plesiochorus*, and comment on the issues of morphological variation within the genus.

## Materials and methods

### Specimen collection

Between December 2019 and October 2021, the urinary and gall bladders of specimens of deceased turtles (*C*. *caretta*, *C*. *mydas*, *E*. *imbricata* and *L*. *olivacea*) were examined during necropsies at Australia Zoo Wildlife Hospital (Beerwah, QLD, Australia) and Dolphin Marine Conservation Park (Coffs Harbour, NSW, Australia). Turtles were collected from multiple localities along the east coast of southern Queensland and northern New South Wales, Australia before transportation to rehabilitation facilities (specific localities listed in relevant taxonomic summaries). Urinary bladders were removed from the host, placed in a Petri dish with 0.85% saline solution, split with a single incision and grossly examined for the presence of trematodes. Gall bladders were examined in combination with liver examination, in which the liver and gall bladder were sliced into sections ~5cm thick, placed into a container and washed with 0.85% saline solution. The solution was left to settle, and three-quarters of the supernatant was discarded. If needed, clean 0.85% saline solution was added to the wash container, left to settle, and the supernatant was discarded again. When sufficiently clean, the sediment was examined in a Petri dish under a dissection microscope. Trematodes were killed and fixed without pressure in near boiling 0.85% saline solution and immediately transferred to 70% ethanol for parallel morphological and molecular characterisation.

### Morphological analysis

Specimens for morphological analysis were washed with fresh water, stained with Mayer’s haematoxylin, destained in 1.0% HCl solution, neutralised in 0.5% NH_3_ (aq), dehydrated in a graded ethanol series, cleared with methyl salicylate, and permanently mounted in Canada balsam on microscope slides. Measurements of specimens were taken using cellSens Standard imaging software (Olympus, Tokyo, Japan) with an Olympus SC50 digital camera mounted on an Olympus BX-53 compound microscope. All measurements are in micrometres (µm) and are presented as a range with the mean in parentheses. Drawings of specimens were completed with the aid of a drawing tube mounted to the same Olympus BX-53 compound microscope, and digitalised with Adobe Illustrator CC 2022.

Morphometric data for samples collected in the present study were natural log-transformed and examined using a principal components analysis (PCA) of the covariance matrix in RStudio v.1.3.959 (http://www.rstudio.com/), and visualised using the package ggfortify (Tang & Horikoshi, 2016). Features included in the PCA are noted in Table 1. Specimens were assigned to a species in the PCA by either i) genetic data from hologenophores included in the PCA or ii) whole worms from *C*. *caretta* were inferred to represent *P*. *cymbiformis* as extensive sequencing showed just one species present in that host.

**Table 1:** Morphometric data from previous studies and the present collection for *Plesiochorus cymbiformis* and *Plesiochorus irwinorum* **n. sp.** expressed as a range and mean in micrometres or as percentages.

	*Plesiochorus cymbiformis*					*Plesiochorus elongatus*		*Plesiochorus irwinorum* **n. sp.**
	Pratt (1914)	Caballero (1954)	Blair and Limpus (1982)	Santoro and Morales (2007)	Present study	Pigulevsky (1953)	Werneck et al. (2019)	Present study
Body length^a^	6000	2500–3200	2970–7210 (5470)	12800–15700 (14000)	4459–9553 (7401)	12000	6800–7700 (7300)	5619–9689 (8398)
Body width^a^	3000	1210–1270	1310–3700 (2200)	4500–5400 (5100)	1695–3877 (2905)	2400	2500–3300 (2800)	2114–4211 (3415)
Length as proportion of width					2.06–3.04 (2.56)			1.79–2.72 (2.48)
Oral sucker length^a^	650	130–240	410–860 (620)	840–1071 (960)	443–863 (713)	770	660–890 (740)	600–900 (826)
% of body length					8–10.85 (9.75)			9.05–10.78 (9.9)
Oral sucker width^a^	650	280–290	420–850 (640)	1140–1323 (1242)	473–933 (777)	770	740–880 (810)	661–996 (886)
Oesophagus length^a^		38–57		315–567 (441)	204–487 (330)		210–300 (250)	197–426 (297)
% of body length					3.06–5.76 (4.5)			2.59–5.01 (3.56)
Oesophagus width^a^		38–46			67–185 (100)		80–120 (98)	85–135 (114)
Pharynx length^a^	800	68–76	150–300 (210)	252–378 (326)	186–301 (258)		230–290 (260)	241–308 (281)
% of body length					2.85–4.18 (3.54)			2.87–4.29 (3.4)
Pharynx width^a^	700	99–100	180–370 (250)	360–378 (376)	223–384 (321)		260–300 (280)	224–376 (331)
Dextral caeca length					3731–8292 (6231)			5576–8455 (7214)
% of body length					73.44–82.85 (88)			77.73–99.23 (88.31)
Dextral caeca width		76–114			88–352 (172)		90–140 (120)	145–305 (213)
Sinistral caeca length					3836–8358 (6333)			5240–8261 (7165)
% of body length					77.4–89.9(77.4)			80.38–93.25 (87.37)
Sinistral caeca width		76–114			94–368 (184)		90–140 (120)	118–334 (214)
Post-caecal length^a^					243–767 (480)			160–1399 (619)
% of body length					3.92–9.45 (6.52)			2.85–14.44 (7.02)
Ventral sucker length^a^	1000	370–390	500–1260 (950)	1512–1890 (1720)	748–1479 (1186)	1300	1100–1800 (1400)	967–1516 (1333)
% of body length					13.6–18.47 (16.15)			13.92–19.41 (16.05)
Ventral sucker width^a^	1000	400–410	620–1370 (950)	1575–1890 (1739)	727–1464 (1175)	1300	1000–1700 (1200)	968–1536 (1304)
Dextral testis length^a^	1000	270–280	480–1520 (900)	2394–3465 (2835)	753–1983 (1423)		1300–1500 (1400)	1216–2218 (1802)
% of body length					14.49–22.05 (18.91)			19.36–22.93 (21.49)
Dextral testis width^a^	1000	190–200	430–1150 (700)	1575–2142 (1932)	579–1579 (1068)		1000–1200 (1100)	1008–1647 (1408)
Sinistral testis length^a^	1000	260–330	480–1520 (900)	2400–3402 (2905)	693–2098 (1350)		1200–1500 (1300)	1160–2189 (1829)
% of body length					14.18–22.81 (18.12)			20.05–25.27 (21.76)
Sinistral testis width^a^	1000	110–170	430–1150 (700)	1638–2205 (1956)	611–1663 (1050)		1000–1100 (1100)	1030–1678 (1459)
Seminal vesicle length		200–280			1419–3939 (2476)			1419–5015 (3501)
% of body length					25.19–54.29 (33.43)			25.25–55.99 (40.13)
Seminal vesicle width	100	38–57			66–175 (110)			118–212 (157)
Ovary length^a^	600	110–140	230–370 (290)	441–630 (536)	300–531 (376)		500–880 (680)	409–733 (560)
% of body length					3.53–7.15 (5.21)			5.44–7.57 (6.68)
Ovary width^a^	200	84–87	210–370 (290)	378–693 (570)	301–655 (374)		410–810 (590)	360–708 (544)
Mehlis’ gland length		65–95		184–315 (283)	112–278 (160)		240–490 (370)	182–312 (242)
% of body length					1.59–3.10 (2.17)			2.16–3.49 (2.85)
Mehlis’ gland width	260	95–152		189–378 (283)	288–144 (221)		240–540 (420)	222–360 (292)
Dextral vitelline follicle length^a^			190–420 (320)	567–945 (796)	351–641 (511)		450–770 (600)	449–745 (626)
% of body length					5.75–8.79 (6.99)			6.46–8.32 (7.47)
Dextral vitelline follicle width^a^			190–500 (280)	315–360 (116)	277–644 (393)		300–780 (540)	310–502 (394)
Sinistral vitelline follicle length^a^			190–420 (320)	567–882 (731)	298–620 (501)		350–860 (580)	375–729 (577)
% of body length					5.39–8.41 (6.82)			5.45–7.9 (6.88)
Sinistral vitelline follicle width^a^			190–500 (280)	504–567 (571)	249–609 (367)		480–780 (580)	314–719 (464)
Egg length^a b^	40	42–46	30–47 (38)	32–39 (34)	36–45 (41)	38–40	76–100 (90)	36–43 (39)
Egg width^a b^	27	27–30	22–41 (31)	32	25–35 (33)	34–52	52–85 (67)	28–34 (32)
Pre-genital pore distance^a^		614–797			1199–2257 (1855)		1700–2000 (1800)	1584–2330 (2011)
% of body length					22.72–29.32 (25.31)			20.79–28.19 (24.15)
Prostatic chamber length^a^	180				75–154 (121)			149–232 (184)
% of body length					1.13–2.42 (1.66)			1.65–2.95 (2.23)
Prostatic chamber width^a^	120				122–246 (171)			85–268 (162)
Uterus to body margin^a^					79–220 (132)			43–87 (61)
% of body length					0.92–3.9 (1.86)			0.48–1.46 (0.75)
Seminal receptacle length^a^	600				222–583 (393)			254–726 (543)
% of body length					3.08–8.05 (5.33)			4.52–9.22 (6.45)
Seminal receptacle width^a^	600				286–634 (451)			232–679 (471)
Post-excretory pore distance					101–443 (274)			197–427 (332)
% of body length					1.26–6.25 (3.73)			3.51–4.7 (4.07)
Pre-ventral sucker distance^a^					1309–2482 (2006)			1627–2479 (2164)
% of body length					24.54–30.96 (27.36)			23.61–31.5 (25.99)
Post-ventral sucker distance^a^					2362–5676 (4190)			2949–5994 (4881)
% of body length					51.67–60.23 (56.25)			52.48–61.88 (57.76)
Pre-testicular distance^a^					2350–4724 (3615)			2790–4551 (3803)
% of body length					41.94–54.94 (49.2)			40.57–55.22 (45.58)
Post-testicular distance^a^					1315–3105 (2303)			1175–3233 (2610)
% of body length					27.05–34.89 (30.96)			20.91–36.1 (30.69)
Pre-ovarian distance^a^					2017–4301 (3339)			2768–4155 (3639)
% of body length					41.62–55.92 (45.36)			39.6–49.26 (43.61)
Post-ovarian distance^a^					2006–5046 (3671)			2468–5056 (4160)
% of body length					44.99–53.63 (49.26)			43.92–52.81 (49.29)
Pre-vitelline follicle distance^a^					1908–3836 (3007)			2615–3689 (3212)
% of body length					37.4–44.66 (40.89)			35.24–46.54 (38.63)
Post-vitelline follicle distance^a^					2179–5163 (3821)			2523–5264 (4407)
% of body length					45.83–56.94 (51.28)			53.12–55.9 (52.1)

^a^Feature used in Principal component analysis ^b^ Eggs measured just posterior to genital pore in this study

Species delineation was based on an integrative interpretation of morphological and genetic data, following the criteria of trematode species recognition proposed by Bray et al. (2022) (i.e. reciprocal monophyly in the most discriminating available molecular marker (here, ITS2 and *cox*1) + distinction in morphology and/or host range).

### Molecular sequencing

Total genomic DNA was extracted from hologenophores cut so as to not interfere with any measurements (Pleijel et al., 2008) using standard phenol/chloroform extraction techniques described by Sambrook & Russell (2001). Following recommendations of Blasco-Costa et al. (2016), three regions were characterised: two nuclear ribosomal DNA (rDNA) regions, the complete second internal transcribed spacer region (ITS2 rDNA) and the partial D1-D3 regions of the large ribosomal subunit region (28S rDNA); and one mitochondrial DNA (mtDNA) region, a partial cytochrome c oxidase subunit (*cox*1 mtDNA) region. Amplification of the ITS2 and 28S regions were completed as described by Cutmore et al. (2016), using the primers 3S (5′-GGT ACC GGT GGA TCA CGT GGC TAG TG-3′; Morgan & Blair, 1995) and ITS2.2 (5′-CCT GGT TAG TTT CTT TTC CTC CGC-3′; Cribb et al., 1998) for the ITS2 region, and the primers LSU5 (5′-TAG GTC GAC CCG CTG AAY TTA AGC A-3′; Littlewood, 1994) and 1500R (5′-GCT ATC CTG AGG GAA ACT TCG-3′; Snyder & Tkach, 2001) for the 28S region. Amplification of the *cox*1 region was performed as described by Wee et al. (2017), using the primers Dig_cox1Fa (5′-ATG ATW TTY TTY TTY YTD ATG CC-3′; Wee et al., 2017) and Dig_cox1R (5′-TCN GGR TGH CCR AAR AAY CAA AA-3′; Wee et al., 2017). Sanger sequencing of purified DNA was performed at the Australian Genome Research Facility, using ABI Big Dye™ v.3.1 chemistry following the manufactures protocols. Sequencing of the *cox*1 and ITS2 regions were completed using the same primers as PCR amplification, whereas sequencing of the 28S region was completed using the internal primers 300F (5′-CAA GTA CCG TGA GGG AAA GTT G-3′; Littlewood et al., 2000) and ECD2 (5′-CCT TGG TCC GTG TTT CAA GAC GGG-3′; Littlewood et al., 1997). Geneious Prime® 2021.0.3 (https://geneious.com) was used to assemble and edit contiguous sequences.

### Phylogenetic analysis

Newly generated *cox*1 and ITS2 sequences were aligned separately with sequences from GenBank (Table 2) using MUSCLE in MEGAX (Kumar et al., 2018), with UPGMA clustering for iterations 1 and 2. Indels greater than three base pairs affecting >5% of sequences were removed. To determine the correct reading frame in the *cox*1 dataset, the aligned data was translated in MESQUITE v.3.6 (Maddison & Maddison, 2021) using the echinoderm/flatworm mitochondrial code, inspected for stop codons and trimmed to start on position one. All three codons were tested for substitution saturation in DAMBE7 (Xia et al., 2003; Xia & Lemey, 2009; Xia, 2018), with no significant substitution saturation detected. Neighbour-joining (NJ) analyses were conducted independently on the aligned *cox*1 and ITS2 datasets in MEGA X to determine species identities. The parameters for the NJ analyses were: “test of phylogeny = bootstrap”, “no. of bootstrap replications = 10,000”, “model/method = No. of differences”, “substitutions to include = d: Transitions + Transversions” and “rates among sites = Uniform rates”. Pairwise distance matrices were generated in MEGA X to determine intra- and interspecific variation within the *cox*1 and ITS2 datasets. The parameters used for the pairwise distances matrices were the same as those used in the NJ analyses. *Phyllodistomum pacificum* Yamaguti, 1951 and *Phyllodistomum vaili* Ho, Bray, Cutmore, Ward & Cribb, 2014 were designated as the function outgroup in the ITS2 NJ analysis based on a preliminary analysis of the 28S dataset.

**Table 2:** ITS2 and 28S sequence data for species of the Gorgoderidae used in ML, BI and NJ analyses.

Species	Host	Location	28S	ITS2	Reference
**Gorgoderidae**					
**Anaporrhutinae**					
*Anaporrhutum* sp.	*Chiloscyllium punctatum*	Queensland, Australia	KF013184	KF013159	Cutmore et al. (2013)
*Nagmia floridensis*	*Rhinoptera bonasus*	Mississippi, USA	AY222262		Olson et al. (2003)
*Nagmia* sp.	*Stegostoma fasciatum*	New South Wales, Australia	KF013192	KF013158, KF013168	Cutmore et al. (2013)
*Plesiochorus cymbiformis*	*Caretta caretta*	Brazil		KF578463	Domiciano et al., Unpublished
		USA		KC494054	Stacy, Unpublished
*Plesiochorus elongatus*	*Caretta caretta*	Rio de Janeiro, Brazil		MK577499–501	Werneck et al. (2019)
*Plesiochorus* sp.	*Caretta caretta*	Off Virginia, USA	KF013180	KF013154	Cutmore et al. (2013)
*Staphylorchis cymatodes*	*Chiloscyllium punctatum*	Moreton Bay, Australia	HM486318	HM486321–23	Cutmore et al. (2010)
**Degeneriinae**					
*Degeneria halosauri*	*Halosauropsis macrochir*	NE Atlantic Ocean	AY222257		Olson et al. (2003)
**Gorgoderinae**					
*Gorgoderina cygnoides*	*Pelophylax ridibundus*	Kokaljane, Bulgaria	AY222264		Olson et al. (2003)
*Gorgoderina lufengensis*	*Nanorana yunnanensis*	China	MH277507		Ding, Unpublished
*Gorgoderina* sp.	*Rana* sp.	Mexico	HQ325007		Rosas-Valdez et al. (2011)
*Phyllodistomum angulatum*	*Sander lucioperca*	Volga River, Russia	KX957735		Stunžėnas et al. (2017)
*Phyllodistomum brevicecum*	*Umbra limi*	Manitoba, Canada	KC760204		Razo-Mendivil et al. (2013)
*Phyllodistomum centropomi*	*Centropomus parallelus*	Veracruz, Mexico	KM659384		Pérez-Ponce de León et al. (2015b)
*Phyllodistomum cribbi*	*Zoogoneticus quitzeoensis*	Michoacan, Mexico	KT376722		Pérez-Ponce de León et al. (2015a)
*Phyllodistomum folium*	*Pisidium amnicum*	Loo Jõgi River, Estonia	KJ729541		Petkevičiūtė et al. (2015)
*Phyllodistomum hoggettae*	*Plectropomus leopardus*	Lizard Island, Australia	KF013191		Cutmore et al. (2013)
*Phyllodistomum hyporhamphi*	*Hyporhamphus australis*	Moreton Bay, Australia	KF013190		Cutmore et al. (2013)
*Phyllodistomum inecoli*	*Profundulus* sp.	Oaxaca, Mexico	KM659389		Pérez-Ponce de León et al. (2015b)
*Phyllodistomum kanae*	*Hynobius retardatus*	Hokkaido, Japan	AB979868		Nakao (2015)
*Phyllodistomum lacustri*	*Ameiurus melas*	North Dakota, USA	EF032692		Curran et al. (2006)
*Phyllodistomum macrocotyle*	*Dreissena polymorpha*	Lake Lukomskoe, Belarus	AY281127		Stunžėnas et al. (2004)
*Phyllodistomum magnificum*	*Tandanus tandanus*	Queensland, Australia	KF013186		Cutmore et al. (2013)
*Phyllodistomum pacificum*	*Pantolabus radiatus*	Moreton Bay, Australia	MG845599	MG845601	Cutmore & Cribb (2018)
*Phyllodistomum parasiluri*	*Silurus asotus*	Japan	LC002522		Urabe et al. (2015)
*Phyllodistomum pseudofolium*	*Pisidium amnicum*	Volga River, Russia	KX957730		Stunžėnas et al. (2017)
*Phyllodistomum sinopapillatum*	*Profundulus balsanus*	Oaxaca, Mexico	KM659382		Pérez-Ponce de León et al. (2015b)
*Phyllodistomum staffordi*	*Ameiurus melas*	Canada	HQ325027		Rosas-Valdez et al. (2011)
*Phyllodistomum* cf. *symmetrorchis*	*Clarias gariepinus*	Lake Victoria, Kenya	KF013174		Cutmore et al. (2013)
*Phyllodistomum umblae*	*Coregonus albula*	Karelia, Russia	KJ729528		Petkevičiūtė et al. (2015)
*Phyllodistomum vaili*	*Mulloidichthys flavolineatus*	Lizard Island, Australia	KF013173	KF013155	Cutmore et al. (2013)
*Phyllodistomum wallacei*	*Xenotaenia resolanae*	Mexico	KT376714		Pérez-Ponce de León et al. (2015a)
*Pseudophyllodistomum anguilae*	*Tilapia zillii*	China	MG976846		Zhang et al., Unpublished
*Pseudophyllodistomum johnstoni*	*Macrobrachium australiense*	Queensland, Australia	KF013177		Cutmore et al. (2013)
*Xystretrum caballeroi*	*Balistes polylepis*	Guerrero, Mexico	HQ325030		Rosas-Valdez et al. (2011)
*Xystretrum solidum*	*Sphoeroides testudineus*	Florida, USA	KF013188		Cutmore et al. (2013)
**Outgroup**					
**Dicrocoeliidae**					
*Lyperosomum transcarpathicus*	*Sorex minutus*	Zakarpatska, Ukraine	AF151943		Tkach (2001)
*Brachylecithum lobatum*	*Corvus corone*	Záhlinice, Czech Republic	AY222260		Olson et al. (2003)
**Encyclometridae**					
*Encyclometra colubrimurorum*	*Natrix natrix*	Kiev region, Ukraine	AF184254		Tkach (2001)

Newly generated partial 28S sequence data were aligned with sequences of related taxa available in GenBank (Table 2) using MUSCLE version 3.7 (Edgar, 2004) on the CIPRES portal (Miller et al., 2010), with ClustalW sequence weighting and UPGMA clustering for iterations 1 and 2. The resulting alignments were manually refined in MEGAX by removing indels greater than 3 bp and affecting >5% of sequences. The refined 28S dataset was analysed using jModelTest 2.1.10 (Darriba et al., 2012) to estimate the best-fit nucleotide substitution model. The model GTR+I+G was predicted to be the best estimator by the Akaike Information Criterion (AIC), and the closest approximation of this model was used in both the Bayesian inference (BI) and maximum likelihood (ML) analyses. The BI analysis was conducted using Mr Bayes 3.2.7a (Ronquist et al., 2012), and ML analysis using RAxML version 8.2.12 (Stamatakis, 2014), both performed on the CIPRES portal. Nodal support for the ML analysis were generated by performing 1,000 bootstrap pseudoreplicates. The BI analysis used the following parameters: 10,000,000 generations (ngen = 10,000,000) with two runs (nruns = 2) containing four Markov Chain Monte Carlo chains (MCMC chains) (nchains = 4), sampling every 1,000th tree (samplefreq = 1,000), “nst = 6”, “rates = invgamma”, “ngammacat = 4” (default), and “ratepr = variable”. The first 30% of sampled trees were disregarded, “sumt burnin value = 3,000” and “sump burnin value = 3,000”. Members of the Encyclometridae Mehra, 1931 and Dicrocoeliidae Looss, 1899 were designated as the functional outgroup, following the relationships inferred by Olson et al. (2003) and Pérez-Ponce de León & Hernández-Mena (2019).

### Data accessibility

The aligned and trimmed ITS2, *cox*1 and 28S datasets, raw morphometric data, and r code used in this study have been uploaded to the Mendeley data repository at 10.17632/j4hzhpjct9.1.

## Results

### General results

The urinary and gall bladders of seven *C*. *caretta*, 76 *C*. *mydas*, 11 *E*. *imbricata* and one *L*. *olivacea* were examined. Specimens conforming morphologically to the genus *Plesiochorus* were found in the urinary bladders of three *C*. *caretta* and two *E*. *imbricata*. No gorgoderids were recovered from the urinary bladders of *C*. *mydas* or *L*. *olivacea*, nor were they recovered from any of the examined gall bladders.

**Figure 1:**
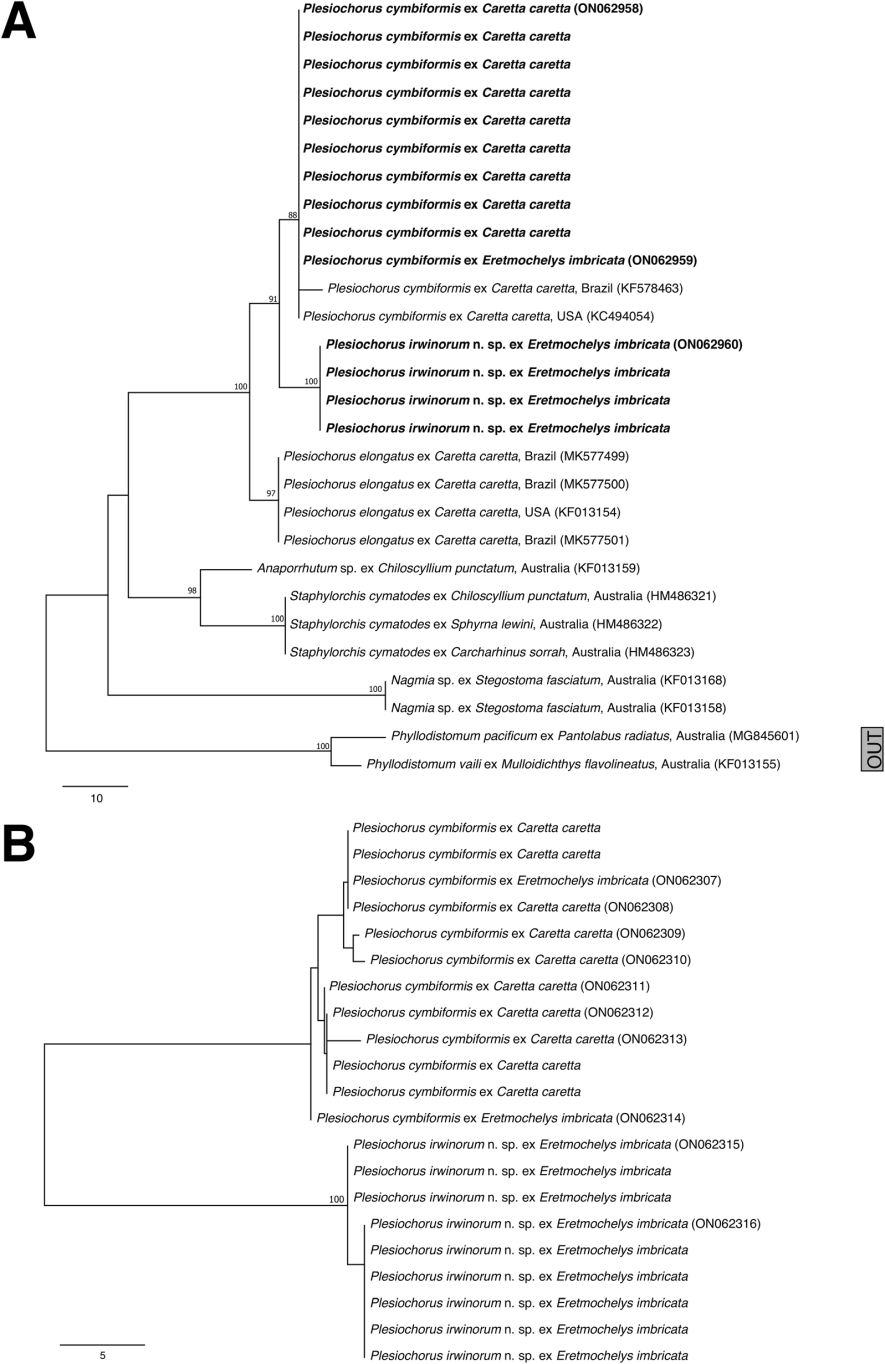
Phylogenetic relationships between anaporrhutine gorgoderids sequenced during this study and those available on GenBank, inferred from (A) rooted neighbour-joining analysis of the ITS2 dataset and (B) unrooted neighbour-joining analysis of the *cox*1 dataset. (A) Taxa in bold represent sequences generated during this study; (B) all sequences in phylogram generated during this study. Bootstrap support is shown above each node; only values >80 are shown. *Scale-bars*: number of base pair differences. OUT, functional outgroup.

ITS2 sequence data were generated for nine individuals from two of the three infected *C*. *caretta*. All newly generated sequence data are 447 bp in length, identical, match sequence data available in GenBank reported for *P*. *cymbiformis* from the USA (**KC494054**), and are similar to sequence data available in GenBank for *P*. *cymbiformis* reported from Brazil (**KC578463**, differing by a single bp) (Fig. 1A). The aligned and trimmed ITS2 dataset yielded 401 bp for analysis. ITS2 sequence data generated for five individuals collected from the two infected *E*. *imbricata* form two clades in the NJ analysis, differing at eight bp (1.7%). One clade matches the new and existing sequence data generated for individuals infecting *C*. *caretta*, identified as *P*. *cymbiformis*; the second clade does not match any available ITS2 sequence data. ITS2 sequence data for new collections of *P*. *cymbiformis* differ from that available in GenBank for *P*. *elongatus* by 10 bp (4.1%), while those for the second clade differ from *P*. *elongatus* by 14 bp (5.8%).

*cox*1 sequence data were generated for 10 individuals collected from all three of the infected *C*. *caretta*. The 10 sequences are 474 bp in length, vary by up to five bp, and do not match any sequence data available in GenBank. *cox*1 sequence data generated for 11 individuals collected from the two infected *E*. *imbricata* again are 474 bp in length, form two distinct clades in the NJ analysis (Fig. 1B), differing at up to 8% (38 bp), neither of which match sequence data available in GenBank. Two sequences form a strong clade with the newly generated sequences from *C*. *caretta*, here identified as *P*. *cymbiformis*. The second, more common clade, has intraclade variation of just one base pair. Considering the consistent differences in both the ITS2 and *cox*1 data, we consider the second clade to represent a species new to science and formally describe it below. Additionally, we report the type-species of *Plesiochorus*, *P*. *cymbiformis*, from Australian *C*. *caretta* and *E*. *imbricata*.

**Family Gorgoderidae Looss, 1899**

**Subfamily Anaporrhutinae **Looss, 1901

**Genus *Plesiochorus*** Looss, 1901

**Type-species: *Plesiochorus cymbiformis* (**Rudolphi, 1819**) **Looss, 1901**, by monotypy**

***Plesiochorus irwinorum*** **n. sp.**

*Type-host*: *Eretmochelys imbricata* (L.) (Cheloniidae)

*Type-locality*: off Hervey Bay (25°14'S 152°49'E), Queensland, Australia

*Other localities*: off Noosa (26°20'S 153°05′E), Queensland, Australia

*Site in host*: Urinary bladder

*Prevalence*: two of 11 *Eretmochelys imbricata* (L.) (18.2%)

*Type-material*: Holotype (QM G240060) and eight paratypes (QM G240061–240068) (all hologenophores)

*Representative DNA sequences*: cox1 mtDNA: nine sequences (two submitted to GenBank ON062315–062316); ITS2 rDNA: four sequences (one submitted to GenBank ON062960); 28S rDNA: one sequence (submitted to GenBank ON062963)

*ZooBank registration*: The Life Science Identifier (LSID) for *Plesiochorus irwinorum* n. sp. is urn:lsid:zoobank.org:act:43312A28-C4B9-4E3E-B7ED-7AF0458E262F

*Etymology*: The specific name *irwinorum* is in honour of the Irwin family of Australia Zoo, Queensland, Australia, who established the Australia Zoo Wildlife Hospital to support animal welfare, conservation, rehabilitation, and scientific research.

**Figure 2:**
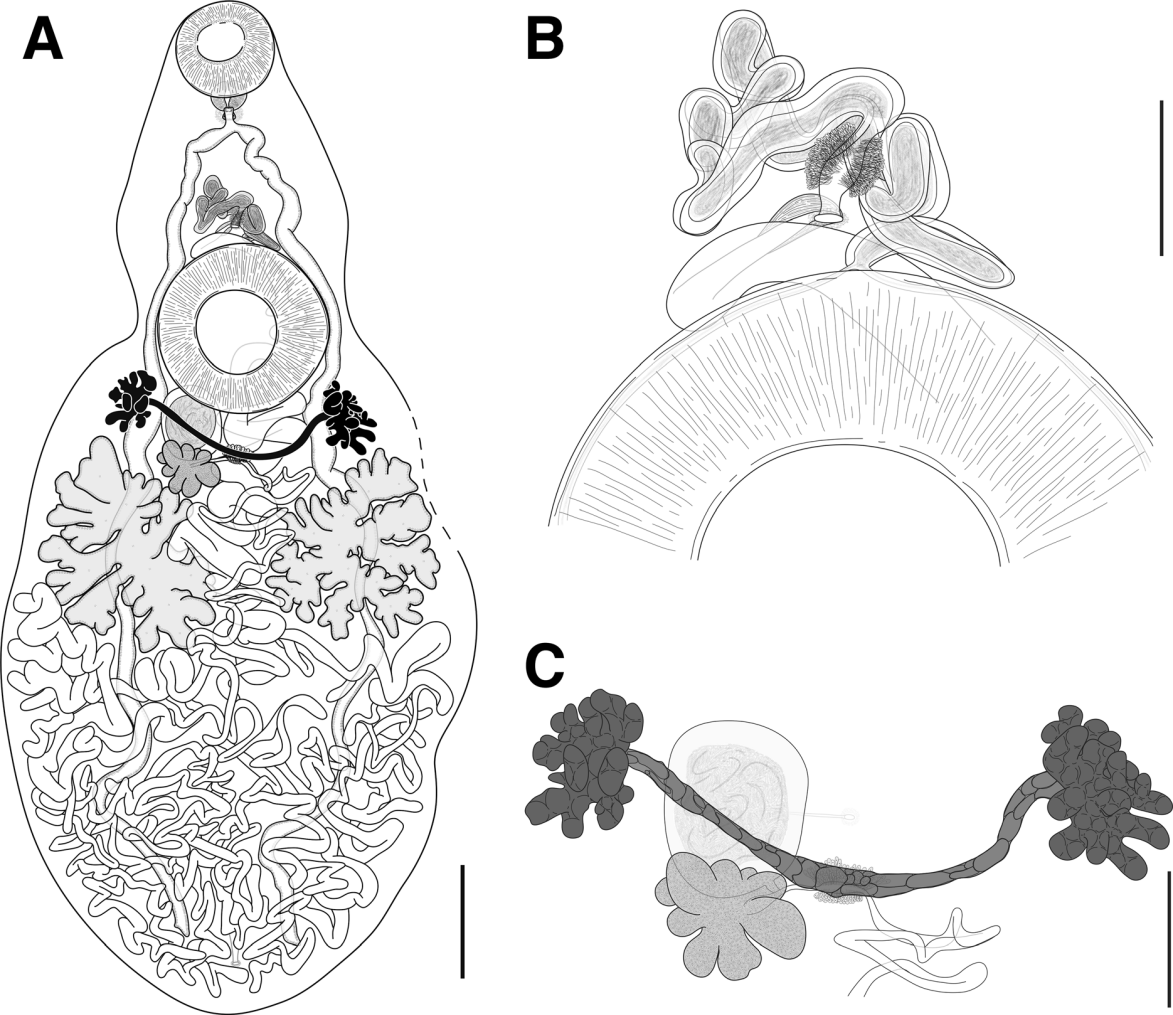
*Plesiochorus irwinorum* **n. sp.** ex *Eretmochelys imbricata*, Holotype. A, Whole worm, ventrally mounted. Dashed line represent section removed for sequencing B, Ventral view of terminal genitalia. C, Ventral view of ovarian complex. *Scale-bars*: A, 1000 µm; B, 200 µm; C, 500 µm.

*Description* (Fig. 2A, B, C)

[Based on nine unflattened, gravid, hologenophores; measurements in Table 1] Functionally cryptic species, not morphologically distinguishable from *P*. *cymbiformis*. Body pyriform to spatulate, aspinous, flattened in dorsoventral plane, widening from just posterior to ventral sucker, widest at level immediately posterior to testes. Posterior end bluntly rounded. Oral sucker round, opening ventro-subterminally. Ventral sucker round, significantly larger than oral sucker. Pharynx spherical or sub-spherical, muscular. Prepharynx not observed. Oesophagus short, often s-shaped in dorsoventral plane. Intestinal bifurcation well anterior to ventral sucker and genital pore. Caeca blind, simple, slightly sinuous, narrow, dorsal to vitellarium, testes, and uterus.

Testes two, large, deeply lobed, generally of similar size, opposite, in middle of body. Vas efferens long, slender, leading from centre of testes to posterior end of seminal vesicle, following margin of ventral sucker. Vas deferens absent. Seminal vesicle elongate, convoluted, anterodorsal to ventral sucker, leading to prostatic chamber. Prostatic chamber a distinct widening of seminal vesicle, surrounded by prostatic cells, ventral to seminal vesicle. Cirrus-sac absent. Ejaculatory duct short. Genital pore medial, ventral, close to anterior margin of ventral sucker.

Ovary deeply lobed, submedial, amphitypic to midline, ventral to uterus and testes when overlapping, mostly anterior to testes, posterior to ventral sucker. Vitellarium in two, symmetrical, deeply lobed, pre-testicular masses, generally of similar size, mostly posterior to ventral sucker, slightly anterior to ovary, ventral to caeca. Mehlis’ gland ovoid, medial to submedial at level of ovary, ventral to uterus. Seminal receptacle roughly spherical to ovoid, anterodorsal to ovary, amphitypic to midline, immediately posterior to ventral sucker. Laurer’s canal dorsal to seminal receptacle, leading to pore on dorsal surface of body. Uterus extensive, filling posterior third of body, intra- and extracaecal posterior to testes, intracaecal anterior to and at level of testes, reaches close to margin of body in large specimens. Eggs ovoid, smallest at formation, gradually increasing in size along uterus, largest just posterior to genital pore.

Excretory vesicle tubular, anterior section obscured by uterus, morphology undetermined. Excretory pore sub-terminal, dorsal, medial, opening dorsally, just posterior to termination of caeca.

***Plesiochorus cymbiformis*** (Rudolphi, 1819) Looss, 1901

*Synonyms*: *Distoma cymbiforme* Rudolphi, 1819, *Phyllodistomum cymbiforme* (Rudolphi, 1819) Braun, 1899, *Spathidium cymbiforme* (Rudolphi, 1819) Looss, 1899

*Type-host*: *Caretta caretta* (L.) (Cheloniidae)

*Type-locality*: Adriatic Sea

*Other hosts*: *Chelonia mydas* (L.), *Eretmochelys imbricata* (L.), and *Lepidochelys olivacea* (Eschscholtz) (Cheloniidae)

*Other localities*: Australia, Brazil, Costa Rica, Cuba, Florida, USA, Gulf of Panama, India, the Mediterranean Sea, Mexico, Morocco, New Guinea, Pakistan, Puerto Rico, the Red Sea

*Site in host*: Urinary Bladder

### New material

*Hosts*: *Caretta caretta* (L.); *Eretmochelys imbricata* (L.)

*Localities*: off Maryborough (25°28'S 152°52'E), and off Hervey Bay (25°14'S 152°49'E), Queensland, Australia

*Site in host:* Urinary bladder

*Prevalence*: three of seven *Caretta caretta* (42.9%); two of 11 *Eretmochelys imbricata* (18.2%)

*Voucher-material*: Six specimens deposited in the QM, four from *Caretta caretta* (G240071–240074) and two from *E*. *imbricata* (G240069–240070)

*Representative DNA sequences*: cox1 mtDNA: 12 sequences (eight submitted to GenBank; six from *Caretta caretta* ON062308–062313; two from *Eretmochelys imbricata* ON062307 and ON062314); ITS2 rDNA: 10 sequences (two submitted to GenBank; one from *Caretta caretta* ON062958; one from *Eretmochelys imbricata* ON062959); 28S rDNA: two sequences (both submitted to GenBank; one from *Caretta caretta* ON062962; one from *Eretmochelys imbricata* ON062961)

**Figure 3:**
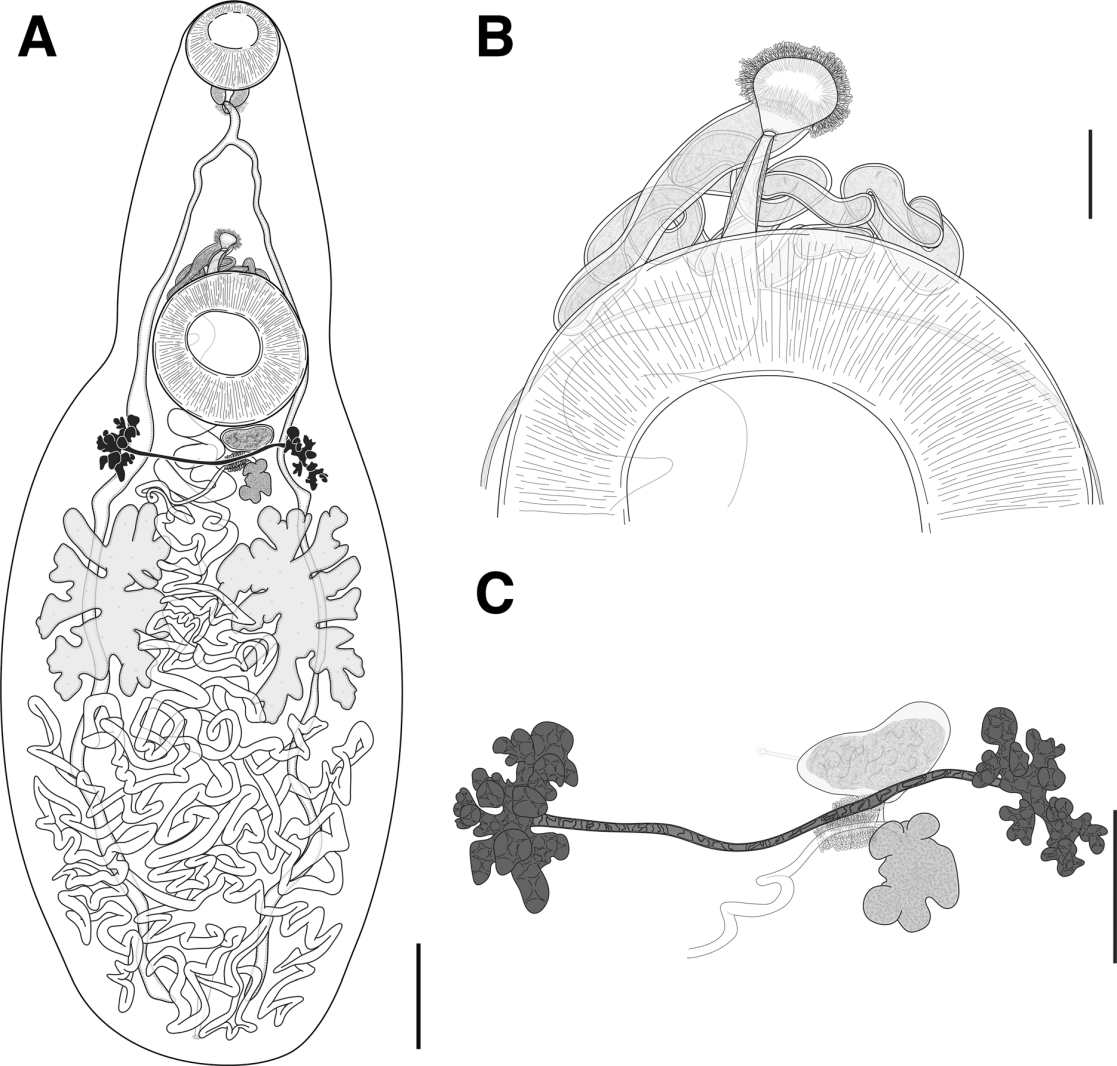
*Plesiochorus cymbiformis* ex *Caretta caretta*. A, Whole worm, ventrally mounted. B, Ventral view of terminal genitalia. C, Ventral view of ovarian complex. *Scale-bars*: A, 1000 µm; B, 200 µm; C, 500 µm.

*Description* (Fig. 3A, B, C)

[Based on 12 unflattened, gravid, hologenophores from *C*. *caretta* and *E*. *imbricata*, and 10 unflattened, gravid, whole worms from *C*. *caretta*; measurements in Table 1.] Functionally cryptic species, qualitatively not morphologically distinguishable from *P*. *irwinorum* **n. sp.** Subtle differences seen only in uterus; does not reach close to body wall.

*Remarks:*

*Plesiochorus cymbiformis* was described by Rudolphi (1819) as *Distoma cymbiforme* Rudolphi (1819), infecting the urinary bladder of *C*. *caretta* from the Adriatic Sea. The original description was vague and contained no morphometric data. Looss (1901) later recombined the species as *Plesiochorus cymbiformis* but did not provide a redescription. The earliest description with morphometric data appears to be that of Pratt (1914) (see Table 1). Subsequent redescriptions of *P*. *cymbiformis* are broadly consistent with those provided by Pratt (1914), although overall body size (and features expected to correlate with body size) vary.

### Morphometric results

**Figure 4:**
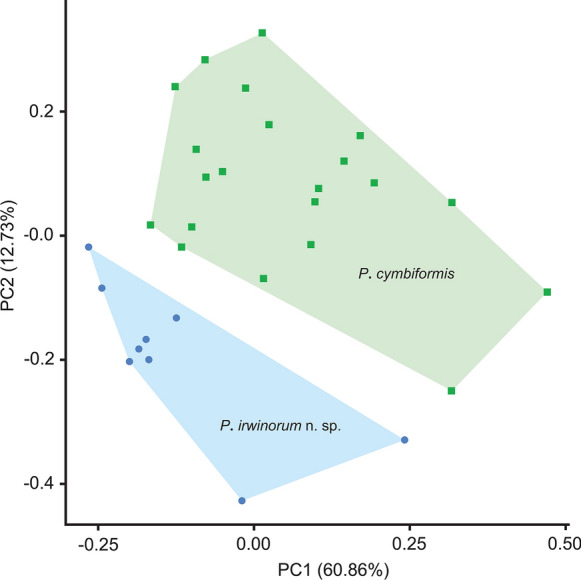
Principle components analysis (PCA) on morphometric data generated from holegenophores and whole worms of *Plesiochorus cymbiformis* (green squares), and hologenophores of *Plesiochorus irwinorum* **n. sp.** (blue circles)

Preliminary analyses of the morphometric data generated for hologenophores from *C*. *caretta* and *E*. *imbricata* and whole worms from *C*. *caretta* during this study suggests that the two species of *Plesiochorus* examined in this study are morphologically indistinguishable, as the two forms are highly similar and all measured features have at least some overlap (Table 1). PCA was employed to determine if the novel species, *P*. *irwinorum* **n. sp.**, detected *via* molecular data, can be distinguished from *P*. *cymbiformis* on the basis of morphology. The two species form two distinct clusters in this analysis (Fig. 4). Principal component (PC) 1 explains 60.86% of the variation in the dataset, with testes length and width acting as the key explanatory variables for this PC, but it should be noted that overall body length, and features strongly correlated with body length (i.e. ventral sucker length and width, oral sucker length and width, post-testicular region, post-caecal length, etc.), also contribute to PC1, although the overall size of the specimens relating to the two species is strikingly similar. PC2 explains 12.73% of the variation present in the dataset, and is largely explained by the distance between the uterus and the body margin, with some additional minor contribution from ovary length and width. However, when examined in isolation, these features (testis length, testis width, distance between uterus and body margin, ovary length, and ovary width) all have overlapping ranges between the two species examined during this study, and further overlap with measurements of *P*. *cymbiformis* and *P*. *elongatus* from previous studies (see Table 1). When examined as a proportion of body length, the distance between the uterus and the body margin almost consistently differentiates the two species, except for the smallest specimen of *P*. *irwinorum* **n. sp.**, which overlaps with the measurements of *P*. *cymbiformis* collected during this study. All other features examined as a proportion of body length overlap between the two species.

### 28S phylogenetic results

**Figure 5:**
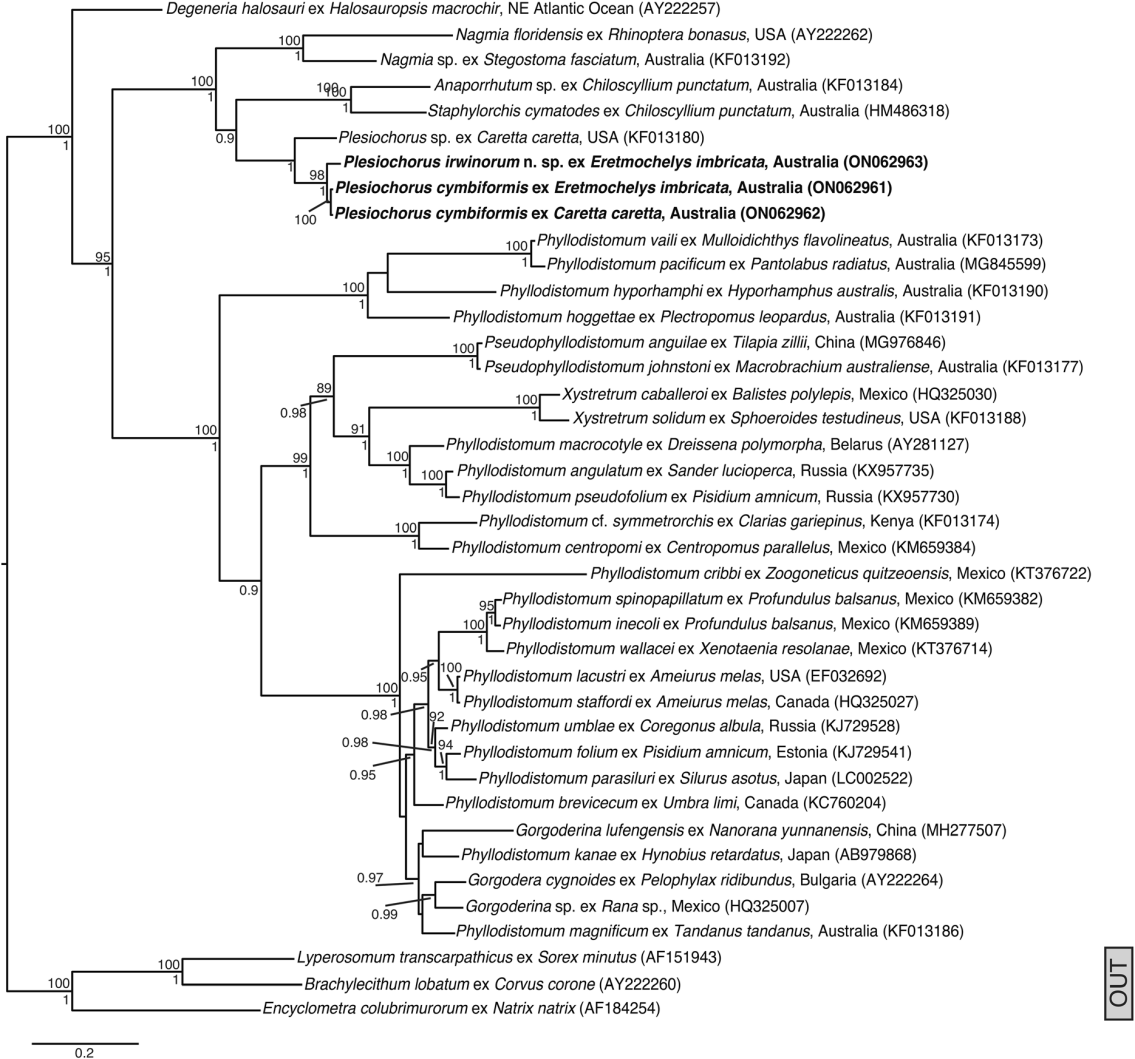
Genetic relationships between species of the Gorgoderidae inferred from 28S rDNA based on maximum likelihood (ML) and Bayesian inference (BI) analyses. Taxa in bold represent sequences from species reported in this study. Posterior probabilities (BI) and bootstrap support (ML) values are shown above and below the nodes, respectively. Nodal support below 0.85 (BI) and 85 (ML) are not shown. *Scale-bar*: expected number of substitutions per site. OUT, functional outgroup.

Alignment of the 28S rDNA dataset yielded 1,154 characters for analysis. The ML and BI analyses produced nearly identical phylograms (Fig. 5), with the only difference seen in the position of *Phyllodistomum cribbi* Pérez-Ponce de León, Martínez-Aquino & Mendoza-Garfias, 2015, which is sister to the clade containing *Gorgodera cygnoides* (Zeder 1800) Zeder 1800, *Phyllodistomum kanae* Nakao, 2015, *Phyllodistomum magnificum* Cribb, 1987, *Gorgoderina lufengensis* Zhang, 2014, *Phyllodistomum folium* (Olfers, 1816) Braun, 1899, *Phyllodistomum umblae* (Fabricius, 1780) Bakke, 1982, *Phyllodistomum lacustri* (Loewen, 1929) Lewis, 1935, *Phyllodistomum spinopapillatum* Pérez-Ponce de León, Pinacho-Pinacho, Mendoza-Garfias & García-Varela, 2015, and *Phyllodistomum brevicecum* Steen, 1938 in the BI analysis, whereas in the ML analysis it forms a polytomy with this clade, with poor support. Members of the Anaporrhutinae form a well-supported clade in both analyses, with *P*. *irwinorum* **n. sp.** forming a well-supported clade with *P*. *cymbiformis*, sister to *P*. *elongatus* (represented by a sequence reported as *Plesiochorus* sp. (**KF013154**), which has corresponding ITS2 data that matches *P*. *elongatus* sequence data generated by Werneck et al. (2019)). In agreement with previous studies (eg. Petkevičiūtė et al., 2020; Pinacho-Pinacho et al., 2021), *Phyllodistomum* Braun, 1899 is paraphyletic in both analyses, with members of *Gorgodera* Looss, 1899, *Gorgoderina* Looss, 1902, *Pseudophyllodistomum* Cribb, 1987, and *Xystretrum* Linton, 1910 forming well-supported clades with species of *Phyllodistomum* within the *Phyllodistomum sensu lato* clade.

## Discussion

### Molecular taxonomy

Both ITS2 and *cox*1 sequence data have been widely and reliably used in the delineation of marine and freshwater gorgoderid species. Closely related, morphologically distinguishable, species of *Phyllodistomum* Braun, 1899 (Gorgoderinae Looss, 1899), such as *Phyllodistomum kupermani* Petkevičiūtė, Zhokhov, Stunžėnas, Poddubnaya and Stanevičiūtė, 2020 and *Phyllodistomum folium* (Olfers, 1816) Braun, 1899 (see Petkevičiūtė et al., 2020), and *Phyllodistomum pseudofolium* Nybelin, 1926 and *Phyllodistomum angulatum* Linstow, 1907 (see Stunžėnas et al., 2017), have been reliably differentiated using ITS2 datasets. Within the Anaporrhutinae, Werneck et al. (2019) showed that specimens of *P*. *elongatus* from Brazil consistently differed from specimens of *P*. *cymbiformis* by 14 base positions in the ITS2 region, while Cutmore et al. (2010) concluded that, despite significant morphological variation, gorgoderids from three orders of elasmobranchs all represented *Staphylorchis cymatodes* (Johnston, 1913) Travassos, 1922 on the basis of identical ITS2 sequence data. Although the differences between *P*. *irwinorum* **n. sp.** and *P*. *cymbiformis* are not large (8–9 bp), the presence of two ITS2 genotypes in complete sympatry (same individual turtle) suggests there is a barrier to genetic exchange between the two species.

*cox*1 mtDNA has also been used for the delineation of gorgoderid species; interspecific variation within morphologically distinct species of the subfamily Gorgoderinae is reported to be as low as 6.2% (19 bp), between *Phyllodistomum spinopapillatum* Pérez-Ponce de León, Pinacho-Pinacho, Mendoza-Garfias and García-Varela, 2015 and *Phyllodistomum inecoli* Razo-Mendivil, Pérez-Ponce de León and Rubio-Godoy, 2013 (see Pérez-Ponce de León et al., 2015b). However, *cox*1 has been shown to vary significantly within species, suggesting that some gorgoderids may have distinct populations, or undescribed cryptic species. Rosas-Valdez et al. (2011) provided evidence that specimens of *Phyllodistomum lacustri* (Loewen, 1929) Lewis, 1935 show significant genetic variation (up to 7.8%) based on geographic and host species separation. They suggested that these data may represent cryptic speciation. The data generated during this study differs from previous reports, in that it explores specimens with highly similar morphology in complete sympatry. The specimens examined during this study, representing *Plesiochorus cymbiformis* and *P*. *irwinorum* **n. sp.**, differ by 8% (38 bp). *cox*1 has limited capacity to recombine (unlike ITS2 and 28S regions, which readily recombine), and genetic differences can accumulate rapidly when two groups of organisms become isolated from each other. This phenomenon can pose a challenge when examining *cox*1 datasets in isolation, particularly when studying parasites which infect hosts with complex movement patterns and populations (Jensen et al., 2019), such as marine turtles. However, when examined in the light of recombining gene data (like ITS2), *cox*1 data can be used to further support taxonomic inferences (Cutmore et al., 2021). Following the species recognition criteria of Bray et al. (2022), the presence of two genetic clades (in the 28S, *cox*1 and ITS2 datasets) from the same geographic region and host species, and indeed in the same host individual, with morphological differentiation (supported by the PCA), does not support the interpretation of a single species with multiple populations, but rather two distinct species, *P*. *cymbiformis* and *P*. *irwinorum* **n. sp.**

### Morphological taxonomy

Based on morphological data in isolation, gorgoderid specimens infecting *E*. *imbricata* during this study appeared to represent a single species, conforming to descriptions of *P*. *cymbiformis*. However, molecular data revealed sufficient genetic differences to consider the specimens to represent two species. The genetic differences are supported by the PCA, indicating the two species can be differentiated on the basis of morphometric data, although the differences do not translate into readily recognisable distinctions.

Significant morphological variation has been documented for some gorgoderids, explored in detail for *S*. *cymatodes* (see Cutmore et al., 2010), *P. folium* (see Namuleno & Scholz, 1994), and *P*. *umblae* (see Bakke, 1988). This phenomenon has also been noted for *P*. *cymbiformis*, and to a smaller extent, *P*. *elongatus* (see Werneck et al., 2019). The most dramatically variable feature for species of *Plesiochorus* is the overall body size, and by extension, features which correlate with it; *P*. *cymbiformis* has been reported between 2.5–15.7 mm in body length, and 1.21–5.4 mm in body width (see Table 1). Specimens collected during the present study also have a significant level of variation within both species, and as a result, measurements of all features overlap between *P*. *cymbiformis* and *P*. *irwinorum* **n. sp.**

Two lines of morphological data were explored to determine if *P*. *cymbiformis* and *P*. *irwinorum* **n. sp.** can be morphologically distinguished. The first, morphometric data in isolation, indicates that the two species are morphologically indistinguishable, as all measured features have overlap between the two species, even when examined as a proportion of body length. The second, PCA, unambiguously distinguishes the two species. A key driving feature for the separation of the two species in the PCA is the distance between the body margin and the uterus, which is, on average, less for *P*. *irwinorum* **n. sp.** (43–87 µm) than for *P*. *cymbiformis* (79–220 µm), especially when examined as a proportion of body length (0.48–1.46% *vs* 0.92–3.9%, respectively). However, there is still overlap in this feature so that it does not reliably distinguish the two species. The PCA also suggests that testes size may distinguish the two species, with *P*. *irwinorum* **n. sp.** generally possessing larger testes as a proportion of body length than *P*. *cymbiformis*; however, there is also overlap between the two species for this feature. Although the PCA did not provide a definitive means of differentiating the two species, the presence of the two, separate clusters further supports the presence of two species. Werneck et al. (2019) also noted that only subtle differences distinguish *P*. *cymbiformis* and *P*. *elongatus*, including a slightly more elongate body and the uterus being closer to the body margin for *P*. *elongatus*. Thus, despite the evidence from the PCA that *P*. *irwinorum* **n. sp.** and *P*. *cymbiformis* are morphologically distinct, the only reliable way to differentiate the three species of *Plesiochorus* is currently by molecular means.

Additional issues surrounding the morphological variation of species of *Plesiochorus* arise when we consider that individuals continue to grow after reaching maturity (as indicated by the possession of a gravid uterus), and the position of various organs (e.g., testis, ovary, uterus) change allometrically. The description of *P*. *irwinorum* **n. sp.** in the present study further shows that morphological differentiation of species within the genus *Plesiochorus* is difficult, ultimately rendering the three species functionally morphologically cryptic. Due to the increasing unreliability of morphological data for the identification of species of *Plesiochorus*, we recommend that future reports be accompanied by molecular data (either ITS2 or 28S, and *cox*1) when possible.

Notably, there are differences between the description of *P*. *elongatus* provided by Werneck et al. (2019) and Pigulewsky (1953), relating mainly to egg size and body shape. Pigulewsky (1953) described specimens infecting *C*. *caretta* from the Adriatic Sea as lanceolate and possessing eggs 38–40 µm in length and 34–52 µm in width (the reporting of eggs being wider than long seems anomalous). The figure of *P*. *elongatus* infecting *C*. *caretta* from Brazil provided by Werneck et al. (2019) is spatulate, rather than lanceolate, and the provided measurements state that they possess eggs almost twice the size (76–100 × 52–85 µm) of those reported by Pigulewsky (1953). However, we think these differences may be sufficient to consider the specimens examined by Werneck et al. (2019) distinct from those reported by Pigulewsky (1953), and warrant further investigation of the voucher material relating to both reports.

### Host specificity

Prior to the present study, no species of *Plesiochorus* has been shown to infect more than one host species on the basis of molecular data; all previous reports of *P*. *cymbiformis* infecting *C*. *mydas*, *E*. *imbricata*, and *L*. *olivacea* are based entirely on morphological data. These reports were all prior to the resurrection of *P*. *elongatus* and occurred when *Plesiochorus* was regarded as monotypic. Given that the three species of *Plesiochorus* are functionally morphologically indistinguishable, we hypothesise that at least some of the previous reports may represent species other than *P*. *cymbiformis*, but without corresponding sequence data, we cannot be certain about their true identities. Nevertheless, the present study provides the first definitive evidence that a single species of *Plesiochorus* can infect two host genera, and we think it is likely that additional sequence data for gorgoderids from *C*. *mydas* and *L*. *olivacea* will provide additional evidence for low host specificity for species of *Plesiochorus*.

Although sequence data are available for all three species of *Plesiochorus*, there is none for the type-host and type-locality for either *P*. *cymbiformis* and *P*. *elongatus* (*C*. *caretta* from the Adriatic Sea). Instead, the available sequence data are from Brazil, the USA (for both *P*. *cymbiformis* and *P*. *elongatus*) and Australia (for *P*. *cymbiformis* and *P*. *irwinorum* **n. sp.**). Thus, we cannot be certain about the genetic identity of *P*. *cymbiformis*, or by extension, *P*. *elongatus*. The certainty of true species identity is further reduced by the fact that both *P*. *cymbiformis* and *P*. *elongatus* infect *C*. *caretta*, and that the sequence data representing both species were generated from this turtle species. It is conceivable that, given the morphological similarities between the two species, the sequence data generated for *P*. *cymbiformis* actually represents *P*. *elongatus*, and *vice versa*, or possibly either or both represent additional, undescribed species. This uncertainty surrounding the true identity for the species of *Plesiochorus* poses an issue when making taxonomic inferences about the genus, and assessing host specificity and geographic distribution. A thorough molecular and morphology-based study of the gorgoderid fauna of *C*. *caretta* of the Adriatic Sea is needed to disentangle the issues surrounding the species identity for species of *Plesiochorus*. Regardless of the true identity of *P*. *cymbiformis* and *P*. *elongatus*, we are confident of the identification of *P*. *irwinorum* **n. sp.**, as the current evidence suggests that it is restricted to *E*. *imbricata*, and is not found in the type-host for *P*. *cymbiformis* and *P*. *elongatus*.
